# Therapeutic effects of diosgenin on alveolar bone loss and apoptosis in diabetic rats with experimental periodontitis

**DOI:** 10.22038/IJBMS.2023.68801.14995

**Published:** 2023

**Authors:** Alper Kızıldağa, Aysan Lektemür Alpanb, Melih Özdedec, Tuğba Aydınd, Özlem Özmene

**Affiliations:** 1 Department of Periodontology, Faculty of Dentistry, Pamukkale University, Denizli, Turkey; 2 Department of Dentomaxillofacial Radiology, Faculty of Dentistry, Dokuz Eylül University, İzmir, Turkey; 3 Department of Periodontology, Faculty of Dentistry, Atatürk University, Erzurum, Turkey; 4 Department of Veterinary Pathology, Faculty of Veterinary Medicine, Burdur Mehmet Akif Ersoy University, Burdur, Turkey

**Keywords:** Alveolar bone loss, Anti-oxidant, Diabetes mellitus, Experimental, Periodontitis, Therapeutics

## Abstract

**Objective(s):**

The present study aims to evaluate the efficacy of administered diosgenin (DG) which has anti-oxidant and anti-inflammatory effects, on alveolar bone loss (ABL) and apoptosis in diabetic rats with periodontitis.

**Materials and Methods:**

Forty male Wistar albino rats (n=40) were divided into five subgroups; control (non-ligated), periodontitis (P), diabetes mellitus (DM), P+DM, and P+DM+DG. To stimulate experimental periodontitis, a ligature was embedded at the gingival margin of the lower first molars for each rat, and diabetes was induced by streptozotocin (STZ) for DM groups. Then, DG (96 mg/kg daily) was performed on the P+DM+DG group by oral gavage for 29 days. At day 30, all animals were euthanized and the distance from the cement-enamel junction to the alveolar bone margin was measured using cone-beam computed tomography as ABL. In addition, immunohistochemical analyses were used to evaluate the expression levels of alkaline phosphatase (ALP), osteocalcin (OCN), bone morphogenetic protein 2 (BMP-2), receptor activator of NF-κB ligand (RANKL), collagen type I (Col-1), B-cell lymphoma-2 (Bcl-2), and Bcl-2-associated X protein (Bax).

**Results:**

Induction of periodontitis and diabetes significantly increased ABL (*P*<0.05). DG administration significantly reduced ABL, expression of RANKL and Bax, and enhanced the expression of ALP, OCN, BMP-2, Bcl-2, and Col-1 in the P+DM+DG group compared with the P+DM group (*P*<0.05).

**Conclusion:**

It is revealed that DG considerably enhanced bone formation and contributed to periodontal healing in this experimental study performed in diabetic rats.

## Introduction

Diabetes is a serious chronic disease that results from insulin insufficiency or deficiency. Poorly controlled or uncontrolled diabetes increases the severity of periodontal disease; hence, periodontitis is recognized as a major complication of diabetes (1, 2). Similarly, severe periodontal disease worsens glycemic control and outcomes in patients with diabetes over time (3-5). 

Periodontitis is a chronic inflammatory disease caused by an imbalance between the resident commensal oral bacterial complex and host-associated oral microbiome dysbiosis. Non-treatment of periodontal inflammation results in the loss of hard and soft tissues due to host-mediated destruction (6). Various mechanisms such as oxidative stress and receptor activator of NF-κB ligand (RANKL) activation play a role in periodontal destruction. In terms of health, reactive oxygen species support the antimicrobial defense system by helping eliminate pathogenic oral bacteria and modulating the gene regulators and cell signaling pathways (7, 8). However, an excessive increase in reactive oxygen species poses a danger to the tissue because reactive oxygen species activate the apoptotic pathways by increasing the expression of B-cell lymphoma-2 (Bcl-2)-associated X (Bax) pro-apoptotic proteins and impair the mitochondria of cells (9). Additionally, high reactive oxygen species levels cause osteoclast cell formation by inducing the expression of RANKL (10). Produced as a membrane-bound or released ligand by activated T- and B-cells, osteoblasts, and fibroblasts, RANKL is a key factor in bone resorption. It promotes osteoclast differentiation and leads to RANKL-related osteoclastogenesis in tissues (10, 11). Gingival crevicular fluid and gingival tissue from patients with periodontal disease demonstrate a higher RANKL expression than those from periodontally healthy patients (12, 13). Several studies linking diabetes with RANKL expression indicated that poor glycemic control provokes RANKL expression (14, 15). Furthermore, diabetes increases the release of RANKL by promoting and prolonging inflammation (15, 16). 

Bone morphogenetic proteins (BMPs), which are growth factors, belong to the transforming growth factor-β superfamily and play a major role in the differentiation, migration, proliferation, and apoptosis of cells. BMP-2 is commonly used to enhance tissue healing in the dental field (17). It modulates the maturation of mesenchymal stem cells into osteoblast cells and thus promotes bone formation (18). Moreover, BMP-2 regulates the maturation of osteoblast cells via secretion of osteocalcin (OCN), osteopontin, alkaline phosphatase (ALP), and collagen type I (Col-1) (19, 20).

Diosgenin (DG) is a naturally occurring steroid saponin and has strong pharmacokinetic effects. In particular, it has anti-oxidant, anti-inflammatory, and glucose-lowering effects (21-23). DG may prevent alveolar bone loss (ABL) by stimulating the signaling of the BMP and Wnt pathways, inhibiting apoptosis, and regulating the expression of RANKL and OCN (24-27). However, despite these properties, no study has yet evaluated the effect of DG in diabetic rats with periodontitis. We hypothesized that DG may promote bone healing and decrease the destructive effects of diabetes and periodontitis on the alveolar bone by regulating oxidative stress, inflammation, and apoptosis. Therefore, we evaluated the effects of DG in a rat model of diabetes and ABL using cone-beam computed tomography (CBCT) and immunohistochemistry.

## Materials and Methods

***Animals***

All experimental procedures were conducted in accordance with the University Ethics Committee for Animal Experiments (PAUHADYEK- 2018/34). The study protocol was created in accordance with the ARRIVE Guidelines (Animal Research: Reporting of *In Vivo* Experiments). Forty male Wistar albino rats (age: 4 months, weight: 350–400 g) were included in the present study. To adapt to the environment, all animals were kept separately in cages under standard laboratory conditions (21±2 °C and 12 hr light:12 hr dark cycle) for 10 days and provided free access to pellet-shaped food and water. The rats were randomly and equally divided into five subgroups: control (non-ligated, n=8), periodontitis (P) (ligature only, n=8), diabetes mellitus (DM) (streptozotocin [STZ] only, n=8), P+DM (ligature+DM, n=8), and P+DM+DG (ligature+DM+DG, n=8). Based on a previous study, DG (Sigma-Aldrich, Saint Louis, MO, USA) was dissolved in distilled water and administered at 96 mg/kg/daily to the P+DM+DG group (26) via oral gavage for 29 days. The animals were sacrificed on the 30^th^ day under general anesthesia.

***Periodontitis***

Periodontitis was induced under anesthesia via administration of 50 mg/kg body weight of ketamine (Eczacibasi Ilac Sanayi, Istanbul, Turkey) and 5 mg/kg body weight of xylazine chloride (Virbaxil, São Paulo, Brazil) intraperitoneally. A sterile silk suture (4-0, Dogsan Ilac Sanayi, Istanbul, Turkey) was embedded in the cervical region of the first lower left and right mandibular molars, and the suture was kept at the submarginal position to induce bacterial and plaque accumulation, inflammation, bone loss, and periodontitis. The same operator (AK) evaluated the ligature positions every day. The first lower left molars were used for CBCT measurements and the first lower right molars for histopathological evaluations.

***Diabetes induction***

The blood glucose level and weight of all animals were determined before the experimental procedure and sacrifice. Experimental diabetes was induced in the DM, P+DM, and P+DM+DG groups using STZ (Sigma-Aldrich). STZ was dissolved in 0.01 M citrate buffer at a pH of 4.5 and injected 50 mg/kg body weight intraperitoneally. Three days after STZ injection, blood samples were obtained from the tail vein, and the blood glucose levels were evaluated using an Accu-Chek Active glucometer (Roche Diagnostics, Mannheim, Germany). The rats with blood glucose levels of >300 mg/dl were considered diabetic (28). The blood glucose levels were measured weekly in the STZ-treated rats throughout the course of the experiment. On day 30, all rats were euthanized via intravenous injection of 150 mg/kg pentobarbital sodium (Oak Pharmaceuticals, Inc., Lake Forest, IL, USA) for tissue collection.

***Imaging procedure***

For three-dimensional (3D) imaging, a supine-positioned CBCT device (Newtom 5G-XL; QR, Verona, Italy) was used. To obtain a better image quality, we selected the smallest field of view (6×6 cm). The exposure conditions were as follows: 100 mm voxel, 110 kV, 11.4 mA, 9.0 sec exposure time, 26.0 s scanning time, enhanced scan, boosted dose, and high-resolution mode. For standardization of imaging procedures, all samples were handled in the same exposure settings and positions. The original NNT software (version 12.1; QR, Verona, Italy) was used to conduct 3D analysis. A 10-year experienced dentomaxillofacial radiologist (MO) blinded to the samples performed all imaging procedures and analyzed the images. ABL was measured from the cemento-enamel junction to the alveolar bone crest, averaging six areas (mesial–medial–distal of the buccal–lingual surfaces) of the first left mandibular molars (Figure 2).

***Histopathological methods***

The obtained right mandibular specimens were fixed in 10% neutral-buffered formalin for histopathological evaluation. The specimens were decalcified in a solution (Osteofast 1, Biognost, Zagreb, Croatia) for 2 weeks, and the solution was changed twice a week. Thereafter, decalcified mandibular tissues were routinely processed using automatic tissue processor equipment (Leica ASP300S; Leica Microsystems, Wetzlar, Germany) and soaked in paraffin. Subsequently, the specimens were cut along the long axis of the tooth in the mesiodistal direction, and serial 5 μm sections were taken using a rotary microtome (Leica RM 2155; Leica Microsystems) from each sample. The 5 μm sections were then stained with hematoxylin and eosin. All histopathological procedures were performed using a light microscope by an experienced researcher (ÖÖ) who was blinded to the study specimen.

Histopathological evaluations were performed at ×40 magnification and in accordance with histopathological scoring criteria. Inflammatory cell infiltration, alveolar bone resorption, and cementum degeneration and destruction were evaluated as previously described (29). score 0: absence of or only discrete cellular infiltration and preserved alveolar process and cementum; score 1: hyperemia, moderate cellular infiltration in the gingival tissue, some but minor alveolar process resorption, and intact cementum; score 2: hyperemia, accentuated cellular infiltration in the gingival/periodontal tissue, accentuated degradation of the alveolar process, and partial destruction of the cementum; and score 3: hyperemia, diffuse cellular infiltration in the gingival/periodontal tissue, ulceration at the gingival epithelium, complete resorption of the alveolar process, and severe destruction of the cementum.

***Immunohistochemical methods***

Following the manufacturer’s instructions, the sections were stained with the following antibodies: BMP-2 (anti-BMP-2 antibody; ab59348, Abcam plc, Cambridge, UK), RANKL (anti-RANKL antibody; ab216484, Abcam plc), ALP (anti-ALP antibody; ab224335, Abcam plc), Bax (anti-Bax antibody; ab53154, Abcam plc), Bcl-2 (anti-Bcl-2 antibody; ab59348, Abcam plc), Col-1 (Col-1 antibody; ab34710, Abcam plc), and OCN (anti-OCN antibody; ab93876, Abcam plc). This procedure was performed using the streptavidin–biotin–peroxidase technique. For all primary antibodies, 1/100 dilution was used.

The Mouse and Rabbit Specific HRP/DAB Detection Kit-Micropolymer (ab236466, Abcam plc) was utilized for the secondary antibody, and 3,3′-diaminobenzidine was used as the chromogen. The primary antibody was disregarded in the negative controls. All immunohistochemical analyses were conducted by a specialized pathologist (ÖÖ) who was blinded to the samples. Seven serial sections were arranged and evaluated for each animal and were scored semiquantitatively by considering the staining intensity as described previously (0, absence of staining; 1, slight staining; 2, medium staining; and 3, marked staining) (19).

Subsequently, computer-assisted histomorphometric and immunohistochemical assessments were performed using an automated image analysis system (Olympus CX41; Olympus Corporation, Tokyo, Japan). The lesioned area was analyzed using the Database Manual CellSens Life Science Imaging Software System (Olympus Corporation).

***Statistical analysis***

The required study sample size was 8 for the groups with periodontitis to reach a statistical power of 90%. The Shapiro–Wilk test was used to measure data normality. Data on ABL were analyzed using the *post hoc* Duncan multiple comparisons test and one-way analysis of variance. The Kruskal–Wallis test along with *post hoc* Mann–Whitney U test with Bonferroni correction was used to compare the independent variables (ALP, Bax, Bcl-2, BMP-2, Col-1, OCN, and RANKL expressions and histopathological scores). Data on ABL were reported as means±SDs (*P*<0.05) and data on immunohistochemical proteins as ranges and medians. All analyses were performed using SPSS version 23 (IBM Corporation, NY, USA).

## Results

***CBCT findings***

**Figure 1:**
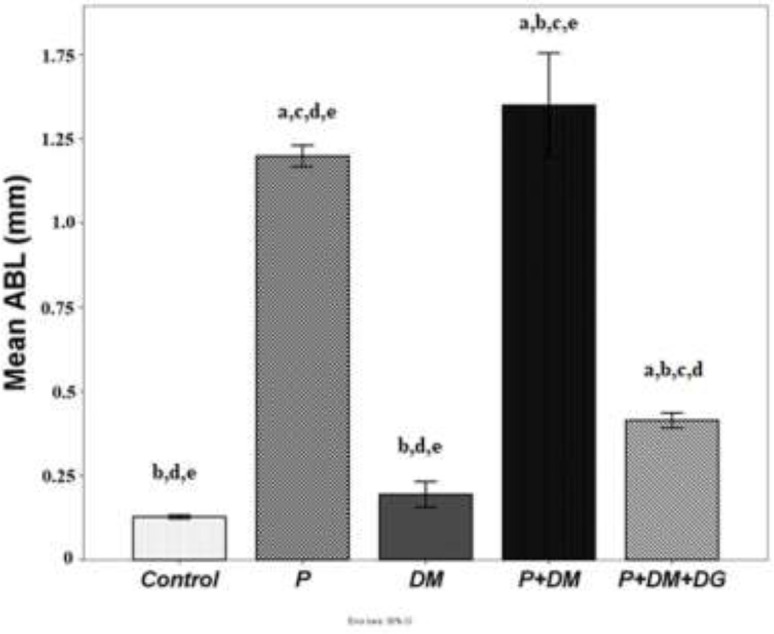
Cone-beam computed tomography measurements of alveolar bone loss of study groups

**Figure 2:**
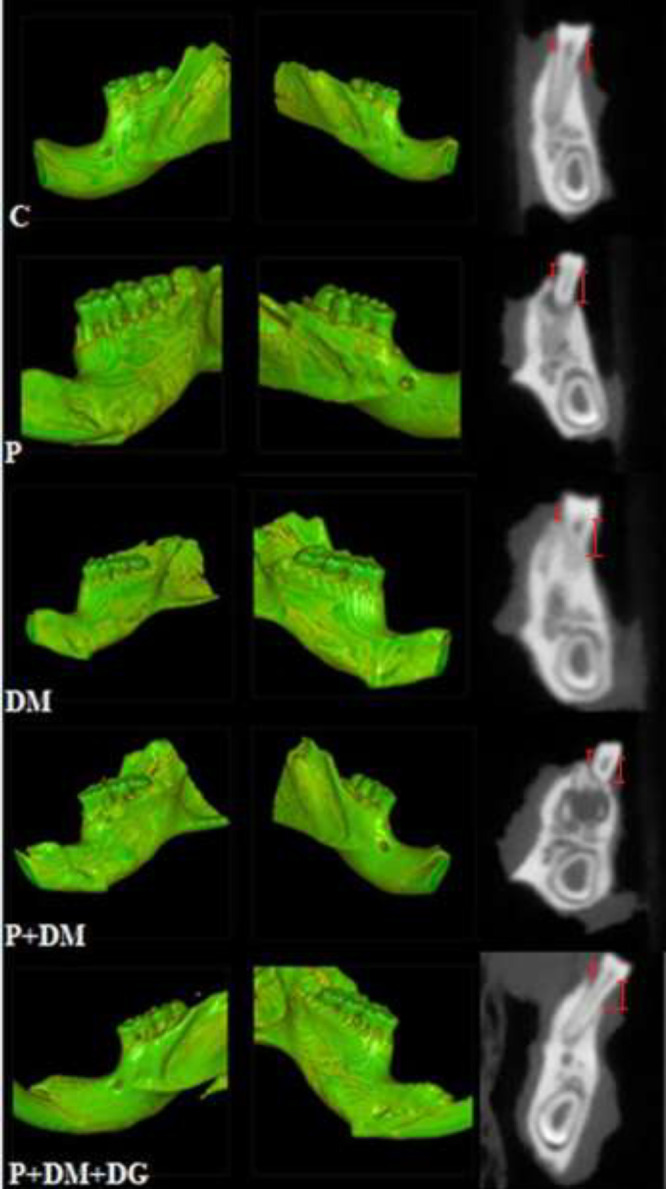
Cone-beam computed tomography images from all study groups

CBCT revealed that the ligature induced ABL in all sutured groups. ABL in the P+DM group was significantly greater than that in the P group (*P*<0.05). ABL was significantly less in the P+DM+DG group than in the P and P+DM groups (*P*<0.05; Figures 1 and 2).

***Histopathological findings***

**Figure 3:**
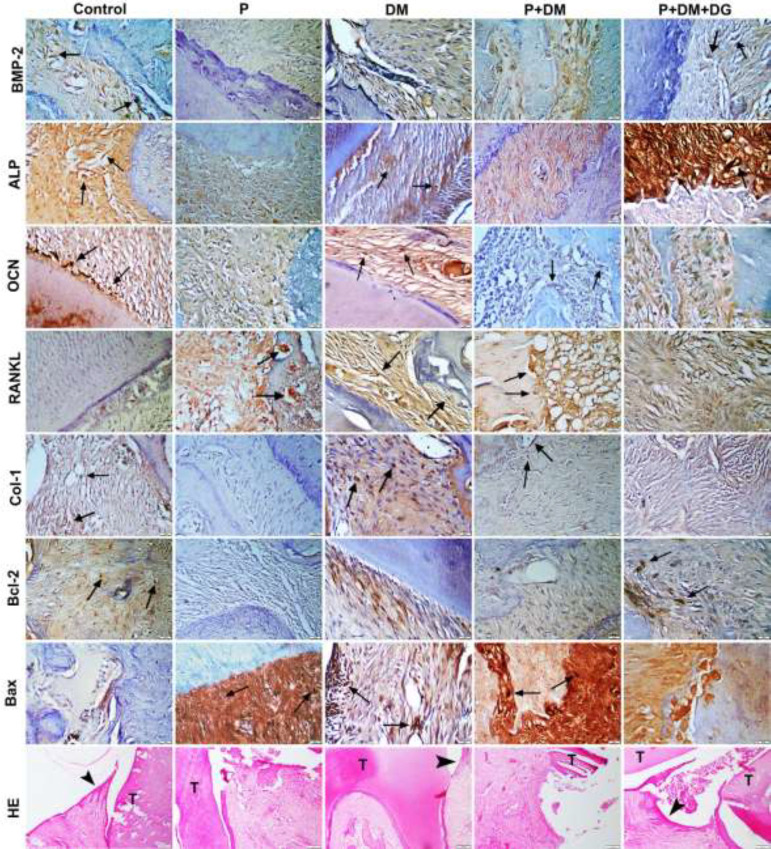
Histopathological sight and immunohistochemical expression of study groups

Normal gingival epithelium and gingival tissue architecture and no pathological findings were detected in the control group. Hyperemia, ulceration at the gingival epithelial layer, inflammatory activities in the gingival and periodontal tissues, partial to severe corruption of the cement, and marked deterioration of the alveolar process were observed in the P, P+DM, and P+DM+DG groups. Meanwhile, the P+DM+DG group showed reduced pathological findings of periodontitis (cellular infiltration in the gingiva and periodontal ligament, alveolar bone destruction, and cement corruption) compared with P and P+DM groups (Figure 3).

***Immunohistochemical findings***

Immunohistochemical staining revealed the expression of ALP, Bcl-2, Bax, Col-1, OCN, RANKL, and BMP-2 in the mesenchymal cells of all groups. Brown color was used to indicate positive immunoexpression. In the control group, a slight to negative expression of ALP, Bcl-2, Bax, Col-1, OCN, RANKL, and BMP-2 was observed (Figure 3).

The expression of ALP, Bcl-2, BMP-2, Col-1, and OCN significantly decreased in the P group compared with that in the control group (*P*<0.05). The P+DM group showed a significantly reduced expression of ALP, Bcl-2, BMP-2, and OCN compared with the DM group (*P*<0.05). In contrast, the P+DM+DG group had a significantly increased expression of ALP, Bcl-2, BMP-2, Col-1, and OCN compared with P and P+DM groups (*P*<0.05). The statistical analysis results are shown in Table 1.

The expression of Bax and RANKL significantly increased in P and P+DM groups compared with that in the control group (*P*<0.05). The P+DM+DG group showed significantly decreased RANKL and Bax expressions compared with P and P+DM groups (*P*<0.05). The expression of RANKL and Bax in all groups is shown in Table 1.

## Discussion

To our knowledge, this study is the first to report the therapeutic effects of DG on ABL in diabetic rats with periodontitis based on histomorphometric, immunohistochemical, and CBCT findings. Several previous studies have evaluated the effects of different doses of DG on bone loss (26, 27). These studies suggested that a high dose of DG significantly modulates bone formation, inhibits bone resorption, and regulates bone metabolism compared with a low dose of DG. Based on a previous study, 96 mg/kg of DG was used to investigate the effect of DG on bone loss in the present study (26).

Micro-computed tomography is considered the gold standard method for the evaluation of periodontal and bone defects. However, it has a high radiation dose and is therefore not performed routinely in clinical practice (30). Thereby, studies have begun to focus on CBCT as an alternative to micro-computed tomography. Kulah *et al*. measured the smallest voxel size using CBCT to evaluate the maxillary trabecular microstructure and detected a significant correlation between micro-computed tomography and CBCT findings (30). ABL has been previously evaluated using CBCT in experimental periodontitis (19). In an *in vitro* study, CBCT was found to be an efficient modality in the assessment of periodontal defects (31). Therefore, CBCT was used to evaluate ABL in the current study.

Type 1 diabetes is a chronic autoimmune disease in which a subclass of T lymphocytes induces apoptosis of pancreatic β cells (32). STZ has been used in several studies to induce diabetes (28). It damages the pancreas and decreases the β cell mass, inducing type 1 diabetes. STZ-induced diabetes is a suitable model for elucidating the mechanisms of diabetes-associated pathogenesis (33). Hence, we used STZ to induce diabetes in this study.

Advanced glycation end products could stimulate apoptosis and oxidative stress in human periodontal ligament cells (34). Furthermore, diabetes and periodontitis significantly increase apoptosis in diabetic rats with periodontitis (28). Bax is a proapoptotic cell and a member of the Bcl-2 family. Bax and Bcl-2 act as stimulators and inhibitors of apoptosis, respectively (19, 28). The expression of Bax and Bcl-2 was then used to evaluate apoptosis in the present study. Herein, the presence of periodontitis and diabetes alone and in combination significantly decreased the expression of Bcl-2 and induced that of Bax. However, DG significantly reduced the expression of Bax and increased that of Bcl-2. DG has also been reported to ameliorate apoptosis in diabetic rats (24). These results suggest that DG may modulate periodontitis and diabetes-related apoptosis by regulating the expression of Bax and Bcl-2 (24).

Several studies have examined RANKL to evaluate the mechanism of the destructive effect of diabetes and periodontitis on the alveolar bone. They demonstrated that periodontitis and diabetes provoke the expression of RANKL and thereby increase alveolar bone destruction (16, 28, 35). Zhang *et al*. (26) showed that DG reduces bone loss in ovariectomized rats by modulating RANKL expression. In the present study, DG significantly decreased the expression of RANKL in the P+DM+DG group compared with that in the P and P+DM groups. Similarly, ABL was significantly reduced in the P+DM+DG group compared with that in P and P+DM groups. Accordingly, DG may prevent bone loss by inhibiting the expression of RANKL, confirming previous findings (26).

The bone continues to form and resorb throughout life. Various growth factors, cytokines, and signaling pathways, including ALP, OCN, Col-1, and BMP-2, are required for the stability of bone metabolism (36). ALP is a critical biomarker of bone formation and can reflect osteoblast cell activity (27). OCN is an essential marker of bone formation and remodeling because it controls mineral deposition (37). Meanwhile, Col-1 promotes osteoblast cell differentiation and mineral matrix accumulation (38). Alpan *et al*. (28) reported that periodontitis and diabetes significantly decreased the expression of ALP, OCN, Col-1, and BMP-2 in rats. Accordingly, we investigated the efficacy of DG in bone metabolism by evaluating the expression of ALP, OCN, BMP-2, and Col-1. A study indicated that DG modulates bone metabolism and hence stimulates bone formation and prevents bone resorption (27). In an *in vitro *study, arginyl–DG conjugate promoted osteoblastic differentiation by inducing BMP-2 and ALP activity (39). Other *in vitro* studies indicated that DG significantly increases the expression of ALP and OCN (40). Our study demonstrated that the DG-treated group showed a significantly increased expression of ALP, OCN, and Col-1 compared with the disease groups. Similarly, BMP-2 has great potential in stimulating bone formation (18). DG modulates the signaling of the Wnt and BMP pathways, which regulate the osteogenic differentiation of mesenchymal stem cells or preosteoblasts. Thereby, DG induces bone formation (25). Herein, the DG-treated group showed a significantly increased BMP-2 expression compared with the disease groups. The present findings suggest that DG stimulates bone formation by enhancing the expression of ALP, OCN, Col-1, and BMP-2, consistent with previous reports (25, 27, 39, 40).

The present study has some limitations, including non-evaluation of the expression of OPG and RANKL/OPG. We also did not assess the total anti-oxidant capacity to examine tissue inflammation and performed only hematoxylin and eosin staining. Additionally, the therapeutic effects of DG on ABL were determined via CBCT analysis only. 

All values were expressed as mean±SD

P: Periodontitis, DM: Diabetes mellitus, DG: Diosgenin

a Statistically significant difference (*P*<0.05) between the control and the others

b Statistically significant difference (*P*<0.05) between P and the others

c Statistically significant difference (*P*<0.05) between DM and the others.

d Statistically significant difference (*P*<0.05) between P+DM and the others

e Statistically significant difference (*P*<0.05) between P+DM+DG and the others

The left and middle columns show three-dimensional reconstruction images of the buccal and lingual areas, respectively; the right column shows cross-sectional images. C: Control P: Periodontitis, DM: Diabetes mellitus, DG: Diosgenin

Normal gingival histology in the control group (arrow); gingival epithelial loss and severe inflammatory reaction in the P group, nearly normal appearance of gingiva in the DM group; severe inflammatory reaction, ulceration, and alveolar bone loss in the P+DM group and decreased inflammatory reaction and periodontal lesions and increased epithelization in the P+DM+DG group. T: teeth; the bars represent 100 µm., P: Periodontitis, DM: Diabetes mellitus

**Table 1:** Statistical analysis results of immunohistochemical scores of study groups

Groups	Control	P	DM	P+DM	P+DM+DG
	min-max (median)	min-max (median)	min-max (median)	min-max (median)	min-max (median)
Histopathological Scores	0−0(0)^b,c,d,e^	2−3(2)^a,c,e^	0−1(1)^a,b,d^	2−3(3)^a,c,e^	1−2(2)^a,b,d^
ALP	1-3 (2)^ b,c,d^	0-1 (0)^ a,c,e^	0-2 (1)^ a,b,d,e^	0-1 (0)^ a,c,e^	1-3 (2)^ a,b,d^
OCN	2-3 (3)^ b,c,d,e^	0-1 (1)^ a,c,e^	1-2 (1)^ a,b,d^	0-1 (0)^ a,c,e^	1-3 (2)^ a,b,d^
BMP-2	0-2 (2)^ b,d,e^	0-1 (0)^ a,c,e^	0-1 (1)^ b,d,e^	0-1 (0)^ a,c,e^	1-3 (2)^ a,b,c,d^
Col-1	2-3 (3)^ b,c,d^	0-1 (0)^ a,e^	0-2 (1)^ a,e^	0-1 (0)^ a,e^	1-3 (2)^ b,c,d^
RANKL	0-1 (0)^ b,c,d,e^	2-3 (2)^ a,c,e^	0-2 (1)^ a,b,d^	1-3 (2)^ a,c,e^	0-2 (1)^ a,b,d^
Bax	0-1 (0)^ b,c,d^	1-3 (3)^ a,e^	1-3 (2)^ a,e^	2-3 (2)^ a,e^	1-2 (1)^ b,c,d^
Bcl-2	1-3 (2)^ b,c,d^	0-2 (1)^ a,c,e^	1-2 (1)^ a,b,d,e^	0-1 (0)^ a,c,e^	1-3 (3)^ b,c,d^

## Conclusion

It can be concluded that as a natural product with anti-inflammatory activities, DG regulates the pathological effects of periodontitis by increasing the expression of BMP-2, ALP, OCN, and Col-1 and reducing the expression of RANKL, apoptosis, and ABL. Future studies need to compare CBCT with micro-computed tomography and evaluate different parameters, including the Wnt/β-catenin pathway, to further elucidate the mechanism of DG.

## Authors’ Contributions

AK and TA designed the study. AK and ALA applied the experimental procedures. MÖ applied radiographic analyses. ÖÖ performed ımmunohistochemical analyses. ALA carried out the statistical analyses. AK wrote the manuscript. 

## Conflicts of Interest

The authors report no conflict of interest.
